# Osteoporotic Vertebral Body Fractures: New Trends in Differential Diagnosis, Bracing and Surgery

**DOI:** 10.3390/jcm11175172

**Published:** 2022-09-01

**Authors:** Panagiotis Korovessis

**Affiliations:** General Hospital “Agios Andreas”, 262224 Patra, Greece; korovess@otenet.gr

In the past, many researchers have investigated the effects of different conservative and operative treatments for Osteoporotic Vertebral Fractures (OVFs). Currently, fresh OVFs are primarily treated by analgesics, bracing, and functional restoration. However, if pain and disability persist for more than 2 months, vertebral body augmentation by means vertebroplasty [VP] or balloon kyphoplasty [BK] with Polymethylmethacrylate (PMMA) is considered. There has been a debate regarding whether the presence of PMMA within the broken vertebral body fragments inhibits the bone’s healing. However, recent literature has been contradictory, indicating a beneficial effect of PMMA on the bone healing and stabilization. Open surgical treatment including traditional open or minimal invasive surgery (MIS) for decompression, reconstruction with mesh cages, and pedicle screw stabilization should be cautiously performed in osteoporotic elderly patients with progressive neurological deficits and/or painful progressive spinal deformity. Medical co-morbidities should be taken into consideration. This series of six recently published excellent papers summarizes the current advances in the primary diagnosis of OVFs, differential diagnosis from traumatic fractures, conservative treatment with brace, operative treatment with VP with PMMA or spinal reconstruction with combined anterior vertebral body reconstruction with cages, and pedicle screw fixation in patients with or without a neurological deficit.

Tsuyoshi Kato et al. [1] in a multicenter RCT in female elderly patients with fresh one-level OVF, compared the preventive effect of rigid versus soft brace on the anterior vertebral body deformity and clinical outcome measures. The three-month rigid-brace treatment neither significantly prevented posttraumatic spinal deformity, nor offered a better quality of life or reduced back pain compared to the soft-brace. This study had several limitations regarding the number of patients required for the study, method of patient selection, kind of fractures treated and patient assignment to each brace.

A retrospective study by Thomas Vordemvenne [2] validated a, elementary method to differentiate OVFs from traumatic A3/AO-type spinal fractures, taking into consideration a morphological analysis provided by a CT-scan. Posterior vertebral body edge morphology and the amount of posterior vertebral body-edge fragment bulging was quantified using a geometric approach. Additionally, the Hounsfield units of the broken vertebral body, the vertebra above, and the vertebra below the fracture were measured. Using the presented method, the authors found a higher sensitivity, specificity, and positive predictive value than those by evaluating the Hounsfield units. The authors reported significant differences in the extent of bulging between osteoporotic and traumatic vertebral fractures, and they have therefore postulated that their bulging analysis method of the dorsal edge fragment in traumatic A3 fractures enables a simple and valid differentiation between the two different kinds of vertebral fractures. 

Naoki Segi et al. [3] retrospectively compared outcomes between MIS via an anterolateral approach (LAVR) using a rectangular footplate cage for vertebral reconstruction (Group L) and the conventional open anteroposterior surgery (group C) with a cylindrical footplate cage in patients aged >56 years suffering from osteoporotic fractures, neurological deficit, severe lower back pain, and pseudarthrosis. The authors showed that the rectangular footplate cage offers superior mechanical support that is derived from the footplate, while the rate of bony fusion was higher than in Group C. The authors underlined the differences that were found both in the fusion rate and fusion morphology. Additionally, the authors postulated that minimal invasive surgery (LAVR) decreases duration of surgery, intraoperative blood loss, respiratory complications, and reduces the loss of correction as compared to conventional open surgery. However, the limitation of LAVR surgery is the limited visualization of the surgical area with less working space. 

A multi-center study by Takumi Takeuci [4] evaluated the roentgenographic results provided by combined 360° surgery with a vertebral body replacement cage (VBR), in patients with previous combined thoracolumbar and lumbar instrumented fusion with a wide footplate expandable cage system. Cage subsidence was observed in 26.3% of cases, whereas the fracture rate of the uppermost instrumented vertebra (UIV) or lowermost instrumented vertebra (LIV) increased in 31.6% of the cases. The authors recommend first posterior and, subsequently, anterior surgery in patients with flexible kyphotic deformity, whereas anterior followed by posterior surgery should be made in patients with rigid kyphosis because of the presence of anterior bony fusion. In patients with rigid kyphosis because of bony bridging between fractured vertebra and adjacent vertebrae, big surgery, including pedicle subtraction osteotomy (PSO) or posterior vertebral column resection, should be selected instead if the surgeon believes that the patient may not benefit from combined anterior and posterior surgery. The authors recommend a combination of posterior more than two levels above, and two below the fixation segments, with VBR to reduce the correction loss and prevent cage subsidence.

The fractured vertebral body union status following VP has not been adequately reported yet, whereas there has been some debate regarding bone healing around the injected PMMA. Despite the effects of immediate PMMA augmentation (e.g., restoration of the lost vertebral body height along with the reduction of anterior vertebral body wedging and immediate stabilization), information about bone healing following VP is contradictory and sparse.

Kawaguchi et al. [5]. first reported that bone generation between the fractured vertebra and the adjacent vertebrae was evident in the patients in their series after VP and further developed to a solidified form during the follow-up observation. Kawaguchi et al. reported on new bone formation in 28.5% of their patients, known as the so called “callus formation”. They believed that callus formation after VP, indicated that PMMA serves as a kind of mechanical stabilizer in the fractured vertebrae. A cadaveric study by Braunstein et al. revealed large amounts of newly formed callus around the injected PMMA, indicating that the new bone around the fractured vertebrae is present following VP [6]. In contrast to Kawaguchi and Brounstein, Buchbinder et al. postulated that there are no evidence-based benefits of VP for osteoporotic VCFs [7]. In contrast, several studies stated that VP is a better choice compared to conservative treatments, regarding pain decrease, improvement in daily activities, and restoration of vertebral body height, as well as the wedging of the fractured vertebral body. However, almost all of these studies are lacking long-term follow-up (>1 year) postoperatively. Yuh-Ruey Kuo [8] in a retrospective study evaluated both new bone formation and the long-term effects after VP in patients with new OCFs who underwent single-level VP for fresh fractures based on a bone scan, CT-scan, or MRI after failed conservative medical therapy. A control group of patients with a single compression fracture, which were treated with analgesics and orthosis, was analyzed for comparison. Comparing the two groups (with/without new bone formation) of patients with VP, 6 months postoperatively, the group with new bone formation showed much more injected PMMA. Yuh-Ruey Kuo [8] showed that the amount of bone cement, the presence of vacuum signs, anterior vertebral compression rate correction, thoracic kyphosis correction, and lumbar lordosis correction might contribute to new bone formation. When comparing the conservative group of treatment and those who received VP, the authors showed significantly higher new bone formation in the VP group at all follow-ups. The control group showed higher vertebral body wedging compared with the VP group. In addition, the restoration of vertebral parameters may contribute to new bone formation. The limitations in this study included that there was no precise time for postoperative radiographs because of different follow-up strategies among surgeons and compliance of patients, while no clinical parameters were investigated. 

There is still a debate as regard to the effect of one level VP on the global spinal alignment. The Yuh-Ruey Kuo [8] study evaluated the changes of global spinal alignment before and following VP and BK in OVCFs. It has been reported that OVC already existed as a kyphotic deformity not only locally but also globally, leading to sagittal imbalance [9]. Studies on the effects of vertebral augmentation on global alignment are increasing, with some of them postulating a potential effect of restoring sagittal spinal alignment. Yokoyama et al. reported that lumbar lordosis increased significantly following BK [10]. Cao et al. reported that lumbar lordosis increased by 2° Cobb after BK and that compression fractures in the thoracolumbar region achieved a better restoration of sagittal balance after percutaneous surgery [11]. Although there was improved alignment after BK, the Yuh-Ruey Kuo results showed that VP did not have a significant effect on the thoracic kyphotic and lumbar lordosis after intervention. Nevertheless, thoracic kyphosis increased with the time lapsed from surgery and became significantly greater than the preoperative value three years postoperatively. The possible theoretical explanation for this phenomenon might be the natural aging process or new remote fractures [11]. It seems from the conclusions of this study that single level VP may have limited influence on the correction of sagittal alignment.

Patients with pathological (osteoporotic, metastatic, hematologic) thoracolumbar fractures (TLF) usually suffer from mechanical back pain, neurological deficits, spinal deformity, and poor quality of life. Traditional open surgery including decompression and stabilization, has been indicated in such patients with progressive papaplegia, cauda equina syndrome, or/and spinal instability. The majority of patients with OVCF who received VP with PMMA showed excellent results on back pain relief rapidly post-intervention even in short-term follow-ups. Some authors explored the long-term mortality and survival after VP or BK performed for pathological OVFs compared with conservative treatment, and they found that BK had better survival than VP, whereas both BK and VP have better survival than the conservative treatment [12,13]. Kuo-Yuan Huang [14] in a retrospective study compared the long-term survival and complications of the patients with osteoporotic and oncologic TL fractures who received VP, BK, open surgery, or conservative treatment. There are only few studies [15] comparing the long-term survival of pathological TL fractures treated with VP, conventional open surgery, and conservative treatment, as well as their related complications (pulmonary embolism, PMMA leakage, shock or death; vertebral infection or osteomyelitis, adjacent vertebral fractures). The authors have questioned whether VP improves long-term survival and reduces complications compared with conventional open surgery and conservative treatment. The initial survival analysis of all patients did not detect any significant difference between VP and conservative treatment. Although there is no difference between VP and conventional open surgery, the 10-year survival of patients who received VP was significantly higher than those who had conservative treatment. Patients with pathological TLFs who had VP seemed to survive longer than those receiving conservative treatment, particularly after the age of 75 years. The limitations of this study were the retrospective design, the choice of treatment based on the fracture type, anatomical factors, health status, etc. Nowadays, there is still a debate [16,17,18] regarding VP, BK, and survival. Although some research showed both VP and BK were better than conservative treatment or sham procedures without cement, and BK may be better than VP in restoring the vertebral height and in possibly providing better survival [16,17], other studies [16,18] did not show consistent results, especially on patients with compression fractures. Although VP is less invasive, and clinical observations found that elderly patients with TL fractures recovered faster after VP with a shorter stay of hospitalization and lower medical expense compared with conventional anterior and posterior spinal decompression or fusion surgeries, Kuo-Yuan Huang [14] did not collect the data on patients’ quality of life and are unable to make any inference on how it improves patient’s function and quality of life.

## References

[1] Kato T., Inose H., Ichimura S., Tokuhashi Y., Nakamura H., Hoshino M., Togawa D., Hirano T., Haro H., Ohba T. (2019). Comparison of Rigid and Soft-Brace Treatments for Acute Osteoporotic Vertebral Compression Fracture: A Prospective, Randomized, Multicenter Study. J. Clin. Med..

[2] Vordemvenne T., Wähnert D., Klingebiel S., Lohmaier J., Hartensuer R., Raschke M.J., Roßlenbroich S. (2020). Differentiation of Traumatic Osteoporotic and Non-Osteoporotic Vertebral AO A3 Fractures by Analyzing the Posterior Edge Morphology—A Retrospective Feasibility Study. J. Clin. Med..

[3] Segi N., Nakashima H., Kanemura T., Satake K., Ito K., Tsushima M., Tanaka S., Ando K., Machino M., Ito S. (2021). Comparison of Outcomes between Minimally Invasive Lateral Approach Vertebral Reconstruction Using a Rectangular Footplate Cage and Conventional Procedure Using a Cylindrical Footplate Cage for Osteoporotic Vertebral Fracture. J. Clin. Med..

[4] Takeuchi T., Yamagishi K., Konishi K., Sano H., Takahashi M., Ichimura S., Kono H., Hasegawa M., Hosogane N. (2022). Radiological Evaluation of Combined Anteroposterior Fusion with Vertebral Body Replacement Using a Minimally Invasive Lateral Approach for Osteoporotic Vertebral Fractures: Verification of Optimal Surgical Procedure. J. Clin. Med..

[5] Kawaguchi S., Horigome K., Yajima H., Oda T., Kii Y., Yoshimoto M., Takebayashi T., Yamashita T. (2010). Conversion to hypertrophic vertebral pseudarthrosis following percutaneous vertebroplasty. Eur. Spine J..

[6] Braunstein V., Sprecher C.M., Gisep A., Benneker L., Yen K., Schneider E., Heini P., Milz S. (2008). Long-term reaction to bone cement in osteoporotic bone: New bone formation in vertebral bodies after vertebroplasty. J. Anat..

[7] Buchbinder R., Golmohammadi K., Johnston R.V., Owen R.J., Homik J., Jones A., Dhillon S.S., Kallmes D.F., Lambert R.G.W. (2015). Percutaneous vertebroplasty for osteoporotic vertebral compression fracture. Cochrane Database Syst. Rev..

[8] Kuo Y.R., Cheng T.A., Chou P.H., Liu Y.F., Chang C.J., Chuang C.F., Su P.F., Lin R.M., Lin C.L. (2022). Healing of Vertebral Compression Fractures in the Elderly after Percutaneous Vertebroplasty—An Analysis of New Bone Formation and Sagittal Alignment in a 3-Year Follow-Up. J. Clin. Med..

[9] Cao Z., Wang G., Hui W., Liu B., Liu Z., Sun J. (2020). Percutaneous kyphoplasty for osteoporotic vertebral compression fractures improves spino-pelvic alignment and global sagittal balance maximally in the thoracolumbar region. PLoS ONE.

[10] Yokoyama K., Kawanishi M., Yamada M., Tanaka H., Ito Y., Kawabata S., Kuroiwa T. (2014). Postoperative change in sagittal balance after Kyphoplasty for the treatment of osteoporotic vertebral compression fracture. Eur. Spine J..

[11] Ailon T., Shaffrey C.I., Lenke L.G., Harrop J.S., Smith J.S. (2015). Progressive Spinal Kyphosis in the Aging Population. Neurosurgery.

[12] McCullough B.J., Comstock B.A., Deyo R.A., Kreuter W., Jarvik J.G. (2013). Major medical outcomes with spinal augmentation vs conservative therapy. JAMA Int. Med..

[13] Ong K.L., Beall D.P., Frohbergh M., Lau E., Hirsch J.A. (2018). Were VCF patients at higher risk of mortality following the 2009 publication of the vertebroplasty “sham” trials?. Osteoporos. Int..

[14] Huang K.Y., Lee S.C., Liu W.L., Wang J.D. (2020). Long Term Survival of Pathological Thoracolumbar Fractures Treated with Vertebroplasty: Analysis Using a Nationwide Insurance Claim Database. J. Clin. Med..

[15] Yang S.Z., Tang Y., Zhang Y., Chen W.G., Sun J., Chu T.W. (2017). Prognostic factors and comparison of conservative treatment, percutaneous vertebroplasty, and open surgery in the treatment of spinal metastases from lung cancer. World Neurosurg..

[16] Chen A.T., Cohen D.B., Skolasky R.L. (2013). Impact of nonoperative treatment, vertebroplasty, and kyphoplasty on survival and morbidity after vertebral compression fracture in the medicare population. J. Bone Jt. Surg. Am..

[17] Lavelle W.F., Khaleel M.A., Cheney R., Demers E., Carl A.L. (2008). Effect of kyphoplasty on survival after vertebral compression fractures. Spine J..

[18] Bae J.W., Gwak H.S., Kim S., Joo J., Shin S.H., Yoo H., Lee S.H. (2016). Percutaneous vertebroplasty for patients with metastatic compression fractures of the thoracolumbar spine: Clinical and radiological factors affecting functional outcomes. Spine J..

